# Amlodipine Vs. hydrochlorothiazide: is there any disparity in its antihypertensive effect or not?

**DOI:** 10.1186/s40885-017-0082-0

**Published:** 2017-12-06

**Authors:** Venu Gopal Jonnalagadda, Kanchan Choudhary, Vijay Kranti Matety

**Affiliations:** 1 Department of Pharmacology & Toxicology, National Institute of Pharmaceutical Education and Research, Bhangagarh, Guwahati, Assam 781006 India; 2 Department of Pharmacology & Toxicology, Bhupal Nobles’ Institute Of Pharmaceutical Sciences, Udaipur, Rajasthan 313001 India

**Keywords:** Hypertension, Calcium channel blockers, Thiazide diuretics, ARB blockers

## Abstract

We have read the article “Comparison of effects between calcium channel blocker and diuretics in combination with angiotensin II receptor blocker on 24-h central blood pressure and vascular hemodynamic parameters in hypertensive patients: study design for a multicenter, double-blinded, active controlled, phase 4, randomized trial” by GC Oh., et al. and found it quite interesting to learn the testing of effectiveness between amlodipine and hydrochlorothiazide in combination with losartan. After 4 weeks of therapy, non-responders were exposed for dose titration of losartan/amlodipine 100/5 mg daily or losartan/hydrochlorothiazide 100/25 mg daily. However, authors didn’t increase in the amlodipine dose from 5 to 10 mg from randomization. However, as per literature both drugs are non-significant in their blood pressure lowering effect. The clarification on above point will further allay the efficacy concerns of clinicians and result in wider usage of future published data.

Dear Editor,

We have read the article **“Comparison of effects between calcium channel blocker and diuretics in combination with angiotensin II receptor blocker on 24-h central blood pressure and vascular hemodynamic parameters in hypertensive patients: study design for a multicenter, double-blinded, active controlled, phase 4, randomized trial”** by GC Oh., et al. and found it quite interesting to learn the testing of effectiveness between amlodipine and hydrochlorothiazide in combination with losartan [1].

In study design, the authors presented that, patients will undergo 4 weeks open labeled run in period followed by eligible patients randomized to either losartan/amlodipine 50/5 mg daily or losartan/ hydrochlorothiazide 50/12.5 mg daily for those who are having mean sitting systolic blood pressure (msSBP) ≥ 140 mmHg. After 4 weeks of therapy, non-responders were exposed for dose titration of losartan/amlodipine 100/5 mg daily or losartan/hydrochlorothiazide 100/25 mg daily. However, authors didn’t increase in the amlodipine dose from 5 to 10 mg.

In the AB Adolphe et al., at the end of week 12, the mean reductions for supine and standing systolic and diastolic blood pressure values with amlodipine 2.5 to 10 mg were −15.2/−12.3 mmHg and −14.0/−11.6 mmHg respectively, as compared to −15.5/−11.1 mmHg and −16.1/−10.1 mmHg after treatment with hydrochlorothiazide25–100 mg with comparable efficacy between both groups [2]. In another study presented by Tanaka N et al., amlodipine and hydrochlorothiazide decreased both systolic and diastolic blood pressure in both groups, but didn’t show any significant difference between both arms after 8 weeks [3].

After analyzing the above data from the study by GC Oh et al.; it was spotted that escalating the dose in one arm cannot signify its superiority over the other. Here in this study the dose for hydrochlorothiazide was escalated from 12.5 to 25 mg whereas the dose for amlodipine lingered same i.e. 5 mg.

Published literatures worldwide are of great help for physicians in going ahead to improve their prescription writing. Their baskets of knowledge are enhanced by these basic findings and hence without maintaining a parallel relationship between the two arms, it will not be reasonable to conclude the superiority of losartan/hydrochlorothiazide over losartan/ amlodipine.

The clarification on above point will further allay the efficacy concerns of clinicians and result in wider usage of future published data.
